# Color in Context: Psychological Context Moderates the Influence of Red on Approach- and Avoidance-Motivated Behavior

**DOI:** 10.1371/journal.pone.0040333

**Published:** 2012-07-11

**Authors:** Brian P. Meier, Paul R. D’Agostino, Andrew J. Elliot, Markus A. Maier, Benjamin M. Wilkowski

**Affiliations:** 1 Department of Psychology, Gettysburg College, Gettysburg, Pennsylvania, United States of America; 2 Department of Clinical and Social Sciences in Psychology, University of Rochester, Rochester, New York, United States of America; 3 Department of Psychology, University of Munich, Munich, Germany; 4 Department of Psychology, University of Wyoming, Laramie, Wyoming, United States of America; University of Florida, United States of America

## Abstract

**Background:**

A basic premise of the recently proffered color-in-context model is that the influence of color on psychological functioning varies as a function of the psychological context in which color is perceived. Some research has examined the appetitive and aversive implications of viewing the color red in romance- and achievement-relevant contexts, respectively, but in all existing empirical work approach and avoidance behavior has been studied in separate tasks and separate experiments. Research is needed to directly test whether red influences the same behavior differently depending entirely on psychological context.

**Methodology/Principal Findings:**

The present experiment was designed to put this premise to direct test in romance- and achievement-relevant contexts within the same experimental paradigm involving walking behavior. Our results revealed that exposure to red (but not blue) indeed has differential implications for walking behavior as a function of the context in which the color is perceived. Red increased the speed with which participants walked to an ostensible interview about dating (a romance-relevant context), but decreased the speed with which they walked to an ostensible interview about intelligence (an achievement-relevant context).

**Conclusions/Significance:**

These results are the first direct evidence that the influence of red on psychological functioning in humans varies by psychological context. Our findings contribute to both the literature on color psychology and the broader, emerging literature on the influence of context on basic psychological processes.

## Introduction

The distinction between approach and avoidance behavior is fundamental to the study of motivation [1], [2]. Lewin [3] characterized this distinction in spatial terms such that approach motivation guides behavior directed at minimizing the distance between oneself and a desired stimulus or event, and avoidance motivation guides behavior directed at enhancing the distance between oneself and an undesired or threatening stimulus or event. In the present research, we examine the link between the color red and approach- and avoidance-motivated behavior, with a particular focus on the moderating role of psychological context. Specifically, we test the hypothesis that exposure to the color red evokes both approach- and avoidance-motivated behavior within the same situation as a function of how that situation is contextualized.

The color red can be a signal of danger, threat, and caution. In some primate species, red coloration on the face or chest signals dominance to potential opponents [4], [5] and is associated with avoidance or withdrawal behavior in conspecifics [6], [7]. In humans, red has long been used as a marker of warning or caution (e.g., stop signs, sirens, the red ink used in grading; [8]) and it serves as an anger cue when viewed on the face or neck [9]–[11]. Empirical work has begun to emerge showing that exposure to the color red has motivational, as well as symbolic, implications for human perceivers. Elliot, Maier, and colleagues [12]–[13] have shown that exposure to red primes avoidance-motivated behavior in achievement situations involving ability evaluation. For example, Elliot et al. [12] found that perceiving the word “items” on a red-colored (versus control-colored) rectangle on the cover of an IQ test led participants to select an easy versus moderately challenging version of the test (see also [14]–[16]).

The color red can also be a signal of sex and romance. In some primates, red coloration on the chest or genitals serves as a signal of sexual receptivity [17], [18] and facilitates approach behavior in potential mates [19], [20]. In humans, red has been associated with passion, romance, and sexuality across the ages in ritual, mythology, literature, and fashion [8], [21], [22]. Again, these associations appear to have more than symbolic implications, as recent research with both men and women has shown that exposure to red enhances their attractiveness ratings of opposite-sex individuals (i.e., evokes approach-related motivation in the romantic realm; [23]–[25].

A color-in-context model has recently been offered as a theoretical account of the link between color and psychological functioning [8], [26]. A core premise of this model is that the influence of color on affect, cognition, and behavior varies as a function of the psychological context in which the color is perceived. Cognitive psychologists have known for some time that color affects perception differently depending on the *physical* context in which it is viewed (e.g., a grey color patch is perceived to be brighter when seen against a darker background; [27]–[29]. However, research is needed to examine whether the same is true regarding the *psychological* context, especially whether red produces different effects in romance- and achievement-relevant contexts. As noted above, some research has examined the appetitive and aversive implications of viewing the color red in these contexts, but in all of this work approach and avoidance behavior have been studied separately – in separate tasks and separate experiments (e.g., rating the attractiveness of opposite-sex targets for appetition in one experiment; selecting an easy versus moderately challenging test for aversion in another experiment). Thus, it is not clear if red can affect the same behavior differently depending entirely on the context in which it is perceived.

In the present experiment, we conducted a strong and direct test of the color-in-context model. We varied the romance- versus achievement-relevance of a situation and sought to determine if the color red would influence approach and avoidance behavior differently in these different contexts. We assessed approach and avoidance behavior using walking speed, which is a direct behavioral indicator of approach-avoidance motivation. We used this same dependent variable in each context, and only varied whether the situation that the participants encountered was characterized as romance-related or ability-related. If the color-in-context model is correct, red should have opposing influences on walking behavior in the two contexts. Specifically, we predicted that participants anticipating an interview with an opposite sex individual in a red shirt on the topic of dating would walk more quickly toward the interview than participants anticipating an interview on the topic of intelligence with the opposite sex individual in a red shirt. No context-based difference in walking speed was predicted when participants anticipated that the interviewer would be wearing a blue shirt. We expected no sex differences in either context. Red has been linked to avoidance motivation and behavior in achievement situations for both sexes (for an exception, see [30]), and red has been linked to approach motivation and behavior in romance situations for both sexes (albeit mediated through different processes, see [24], [31], [32]).

## Methods

### Ethics Statement

All of the research reported herein was approved by the Gettysburg College Institutional Review Board. All individuals gave informed, written consent prior to participating in the experiment. At the end of the experiment, all participants were thoroughly debriefed.

### Participants

Participants were 64 Gettysburg College undergraduate students (22 males; 42 females with an average age of 18.95 years (*SD* = 1.07 years). We did not collect data on participants’ sexual orientation; this was an oversight, and may be considered a limitation of the present research.

### Procedure and Materials

**Figure 1:**
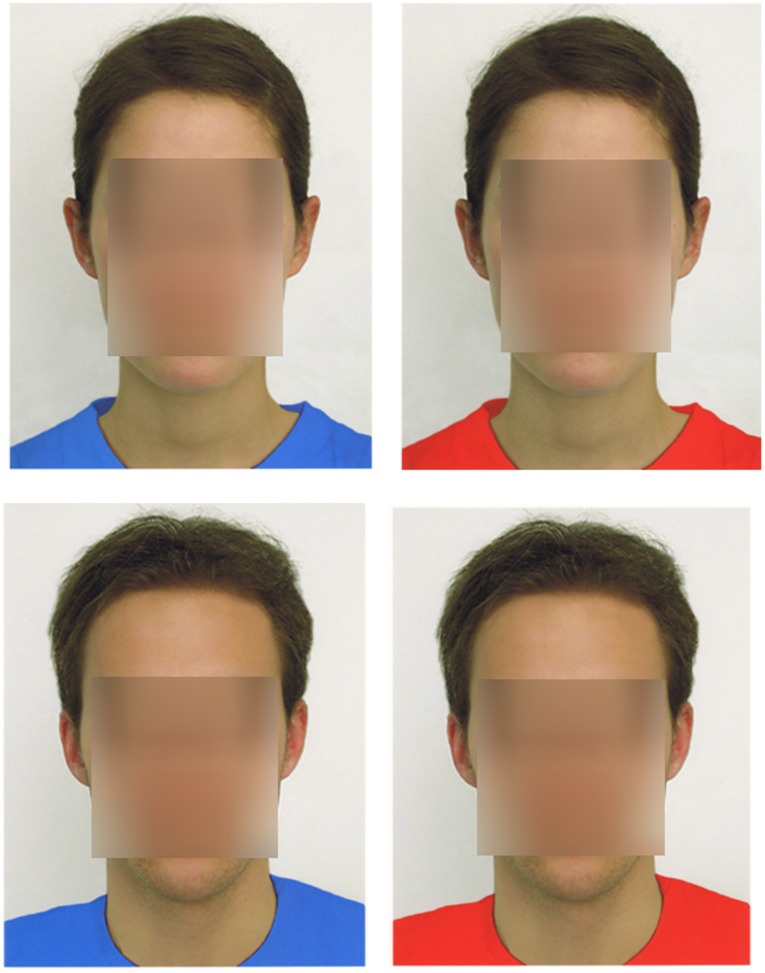
Pictures of “Jackie” and “Jack” in Blue and Red. The faces were intact in the experiment, but are blurred here to protect privacy.

Participants were randomly assigned to one condition of a 2 (context: dating interview or intelligence interview) × 2 (color: red or blue t-shirt) between-participants design. They met a research assistant in a lab room for a study on “motivation,” and were told that they would be interviewed by another research assistant in a separate room “down the hallway.” Participants were told that they would be interviewed about “dating at Gettysburg College” (romance-relevant context) or about “your intelligence” (achievement-relevant context). They were told that there were several interviewers and that we had pictures of all of them so they could see who would be interviewing them later. Then, participants were shown an opposite-sex picture of the person (“Jack” or “Jackie”) who would (ostensibly) be interviewing them; see Figure 1). Each target person was rated as attractive in pilot work [33]. The pictures were 5.5″×4.5″ head and shoulder shots in which the individual was wearing either a red or a blue t-shirt. The pictures were centered on an 8.5″×11″ page and printed on Epson Enhanced Matte archival quality paper with an Epson Stylus Photo R800 printer. We selected red and blue colors that were equal in lightness and chroma using the CIELCh color model and a GretagMacBeth Eye-One Pro spectrophotometer. The parameters for the color red were LCh(51.1/57.7/27.8) and for the color blue were LCh(51.6/57.6/278.3).

Immediately after the color manipulation, participants were taken to the doorway of the initial room and were told to walk down the hallway to room 309 where they would be interviewed. The distance between the first room and the second room was approximately 21 meters. Unbeknownst to participants, a confederate (blind to experimental condition) was seated in the hallway inconspicuously reading. The confederate timed how long it took participants to walk from the first room to the second room. The confederate measured the time in seconds between participants’ first step and the point at which they touched the doorknob of the second room. Participants were debriefed shortly after they entered the second room.

## Results

We conducted a 2 (context: dating interview or intelligence interview) x 2 (color: red or blue t-shirt) between-participants ANOVA on walking speed as measured in seconds. A preliminary analysis including sex as a factor revealed no significant main or interactive effects of sex, so it was omitted from further consideration.

The main effect of context was significant, *F*(1, 60) = 4.56, *p* = .037, partial eta^2^ = .07, indicating that participants walked faster to the interview room when the context was dating (*M* = 16.56; *SD* = 2.86) versus intelligence (*M* = 17.88; *SD* = 2.32). The main effect of color was not significant, *F* <1.

**Figure 2:**
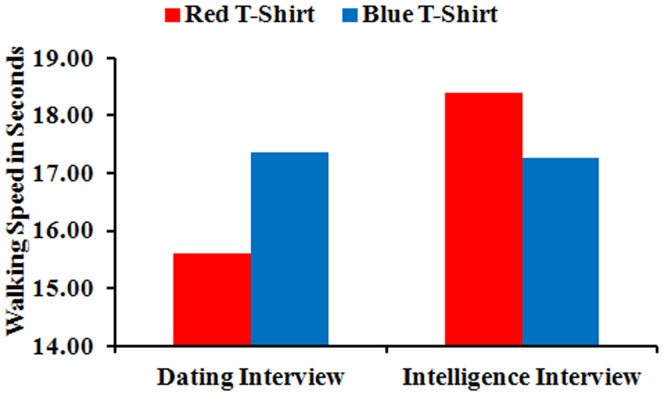
Walking Speed Means for the Context x Color Interaction.

More importantly, as predicted, we found a significant context x color interaction, *F*(1, 60) = 5.07, *p* = .028, partial eta^2^ = .08. As shown in Figure 2, the interaction supported the hypothesis, as participants walked faster to the dating interview when the interviewer was dressed in red (*M* = 15.60; *SD* = 1.57) versus blue (*M* = 17.35; *SD* = 3.44), but slower to the intelligence interview when the interviewer was dressed in red (*M* = 18.39; *SD* = 2.69) versus blue (*M* = 17.27; *SD* = 1.67). This interaction testing the relative effect of color on behavior in two different contexts was our central concern, but we also conducted ancillary analyses on individual effects. These analyses indicated the dating interview versus intelligence interview contrast was significant and in the predicted direction for red, *t*(30) = 3.05, *p* = .001 (one-tailed), *d* = 1.27, and, as anticipated, was not significant for blue, *t*(30) =  −.08, *p* = .47 (one-tailed). Furthermore, the red versus blue contrast was significant and in the predicted direction in the dating interview context: *t*(29) = 1.75, *p* = .046 (one-tailed), *d* = .65, and was marginally significant and in the predicted direction in the intelligence interview context: *t*(31) = 1.40, *p* = .087 (one-tailed), *d* = .50.

## Discussion

The present experiment revealed that red had opposing effects on walking speed as a function of the psychological context in which it was perceived; it increased walking speed in a romance-related context, but decreased walking speed in an achievement-related context. Critically, the opposing effects of red in these different contexts were observed in the same experiment, with the same type of manipulation and materials, and using the same dependent measure, thereby providing direct evidence, for the first time, of context moderation across the romance and achievement domains. Furthermore, our findings are the first to show an effect of any color in any context on walking speed.

Red is a signal in the non-human animal world of both danger and sexual receptivity [5], [20], and there is evidence in a variety of species that red coloration serves different functions in different contexts, including mating and competition contexts (for reviews, see [34]–[36]). The current results extend this work to human behavior by revealing parallel effects to those found “in the wild.” Although our research was not designed to test the ultimate origin or distal cause of these red effects [37], we do think that the clear parallels that may be drawn between human and non-human behavior in this regard hint at a biological basis for the effects [8], [38], [39]. Actually, we suspect that the red effects documented herein are a joint function of biologically-based predispositions that are reinforced and extended by social learning (see [8]). Clearly, however, additional research is needed before definitive statements on this matter may be offered (see [40], for a start at such work).

First and foremost, the present research contributes to the literature on color psychology. However, our research also contributes to the broader psychological literature, especially emerging research on context in social-personality psychology. Context is becoming something of a “hot topic” in this discipline, especially in the areas of emotion, person perception, attitudes, and automatic evaluation [41]–[44]. The present work provides additional impetus for the essential need to attend to context in studying psychological phenomena, as we show that a seemingly incidental environmental cue such as color can have a differential impact on behavior as a function whether it is viewed in a romance- or achievement-relevant context. Parenthetically, a reviewer noted that the achievement-relevant manipulation in our study was somewhat more personally-focused (“your intelligence”) than the romance-relevant manipulation (“dating at Gettysburg College”); in subsequent research using these manipulations, it would be optimal to attend to this discrepancy (perhaps by changing the romance-relevant manipulation to “your dating life at Gettysburg College”).

Exposure to color is a ubiquitous aspect of our moment-to-moment perceptual experience. Nevertheless, psychologists have tended to relegate color to the study of aesthetics or preferences or to downplay it altogether as mere environmental “noise” [45]. The current research joins a small but growing body of work (see [26], for a review) suggesting that color should be more strongly considered in analyses of human behavior. Our results reveal that a subtle manipulation of color can have important effects on basic approach and avoidance behavior and, critically, highlight the importance of attending to context in investigations of color and psychological functioning.
